# Supplementary material for: Performance of VIDAS® Diagnostic Tests for the Automated Detection of Dengue Virus NS1 Antigen and of Anti-Dengue Virus IgM and IgG Antibodies: A Multicentre, International Study
Source: Diagnostics (Basel). 2023 Mar 16;13(6):1137. doi: 10.3390/diagnostics13061137 (PMC10047366; doi:10.3390/diagnostics13061137)
Supplement: Supplementary file 1 [file diagnostics-13-01137-s001.zip › diagnostics-2201429-supplementary.pdf]

Supplementary Materials

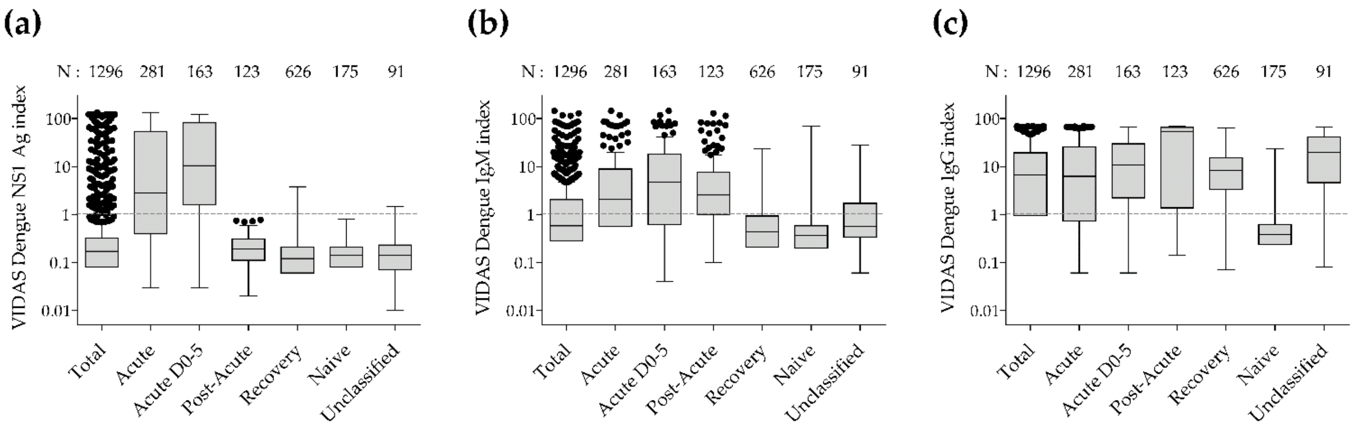

Figure S1. Index values of the VIDAS® dengue assays, in the total population and per DENV infection stage. VIDAS® Dengue NS1 Antigen (**a**), IgM (**b**) and IgG (**c**) assays were performed as described in the Methods section. Index values are depicted as Tukey boxplots in the whole study population (Total) and according to the DENV infection stages (Acute, Acute within 0 to 5 days post symptom onset [D0-5], Post-Acute, Recovery, Naïve and Unclassified), as defined in Table 3. The grey dashed line indicates the positivity cutoff (i = 1.0). The number of samples (N) per group is shown above each box. Respective median (interquartile range) index values are shown in Table S2.

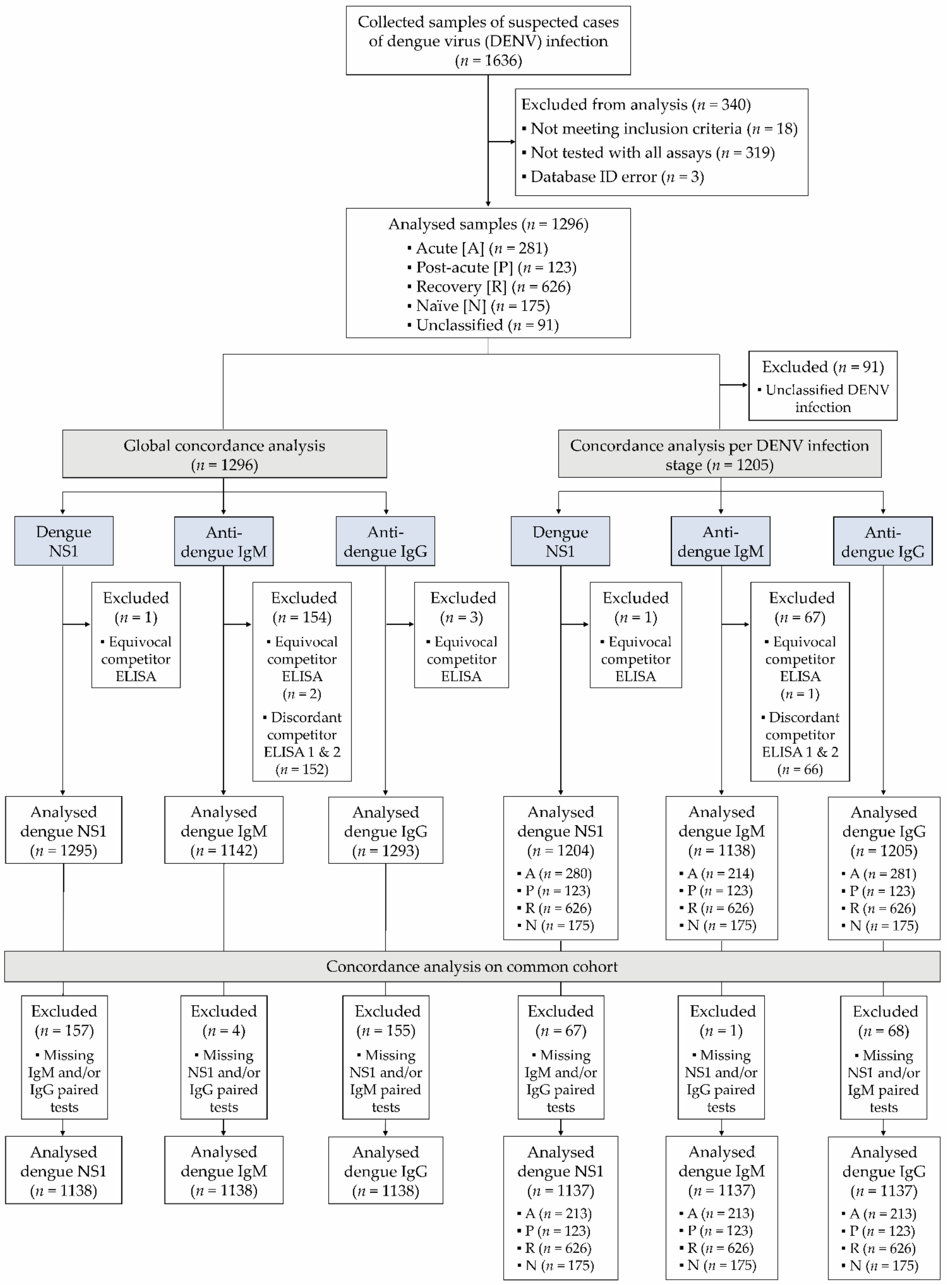

Figure S2. Extended study flow diagram describing the concordance analysis on common samples shown in Table S5.

Table S1. Distribution of samples according to the time from symptom onset, per DENV infection stage.

**Days from symptom onset**	**Stage of DENV infection** [1], ***N*** **(%)**					
	**Acute**	**Post-Acute**	**Recovery**	**Naïve**	**Unclassified**	**Total**
**0 – 3 days**	102 (36.3%)	6 (4.9%)	258 (41.2%)	88 (50.3%)	23 (25.3%)	477
**4 – 5 days**	61 (21.7%)	7 (5.7%)	119 (19.0%)	24 (13.7%)	17 (18.7%)	228
**6 – 8 days**	95 (33.8%)	101 (82.1%)	169 (27.0%)	48 (27.4%)	43 (47.2%)	456
**9 – 15 days**	11 (3.9%)	8 (6.5%)	43 (6.9%)	12 (6.9%)	4 (4.4%)	78
**16 days – 1 month**	7 (2.5%)	0 (0.0%)	23 (3.7%)	2 (1.1%)	2 (2.2%)	34
**> 1 month**	1 (0.4%)	0 (0.0%)	12 (1.9%)	1 (0.6%)	2 (2.2%)	16
**Unknown**	4 (1.4%)	1 (0.8%)	2 (0.3%)	0 (0.0%)	0 (0.0%)	7
**Total**	281	123	626	175	91	1296

[1] As defined in Table 3.

Table S2. Median and interquartile range (IQR) of VIDAS® dengue assays, in the total population and per DENV infection stage (see Figure S1).

VIDAS® dengue assay	variable	DENV infection stage						
		**Total**	**Acute**	**Acute D0-5**	**Post-Acute**	**Recovery**	**Naïve**	**Unclassified**
**NS1**	*N*	1296	281	163	123	626	175	91
	Median	0.2	2.8	10.5	0.2	0.1	0.1	0.1
	IQR	0.1-0.3	0.4-54.1	1.6-81.1	0.1-0.3	0.1-0.2	0.1-0.2	0.1-0.2
**IgM**	*N*	1296	281	163	123	626	175	91
	Median	0.6	2.1	4.7	2.5	0.4	0.4	0.6
	IQR	0.3-2.0	0.6-8.9	0.6-18.0	1.0-7.6	0.2-0.9	0.2-0.6	0.3-1.7
**IgG**	*N*	1296	281	163	123	626	175	91
	Median	6.7	6.2	10.7	53.0	8.2	0.4	19.6
	IQR	1.0-19.2	0.7-25.3	2.2-29.7	1.4-65.3	3.4-15.1	0.2-0.6	4.6-41.5

Table S3. Percentage of positive test results of combined NS1/IgM and IgM/IgG VIDAS® dengue assays *vs.* single assays, in the acute and post-acute stages of DENV infection, respectively.

Population	VIDAS® dengue assay	% positive test results	
		n/N [1]	**% [95% CI]**
**Acute (all)**	NS1	194/281	69.0% [63.4-74.2]
	IgM	175/281	62.3% [56.5-67.7]
	NS1/IgM [2]	233/281	82.9% [78.1-86.9]
**Acute (D0-5)**	NS1	131/163	80.4% [73.6-85.7]
	IgM	108/163	66.3% [58.7-73.1]
	NS1/IgM [2]	141/163	86.5% [80.4-90.9]
**Post-Acute**	IgM	92/123	74.8% [66.5-81.6]
	IgG	99/123	80.5% [72.6-86.5]
	IgM/IgG [3]	113/123	91.9% [85.7-95.5]

[1] *n/N* is the ratio of the number of samples positive with the respective VIDAS® Dengue assays to the total number of samples in the indicated DENV infection stage. [2] Percentage of tests positive for VIDAS® NS1 and/or IgM; [3] Percentage of tests positive for VIDAS® IgM and/or IgG. Abbreviations: CI, confidence interval; D0-5, 0-5 days post symptom-onset.

Table S4. Description of the discordant infection stage classifications between the VIDAS®-based and the competitor ELISA-based algorithm

Stage of DENV infection [3]	Disagreement	VIDAS®-based classification (*n*) [2]				
	n/*N* [1] (%)	Acute	Post-Acute	Recovery	Naïve	Unclassified
**Acute (all)**	32/281 (11.4%)	-	18	4	3	7
**Acute (D0-5)**	7/163 (4.3%)	-	5	1	0	1
**Post-Acute**	45/123 (36.6%)	0	-	21	10	14
**Recovery**	196/626 (31.3%)	5	132	-	46	13
**Naïve**	33/175 (18.9%)	0	9	10	-	14
**Unclassified**	89/91 (97.8%)	1	31	50	7	-

[1] *n*/*N* is the ratio of the number of samples whose classification using the defined rules (Table 3) by the VIDAS® assays was discordant with that by the competitor ELISA, to the number of samples classified by the competitor ELISA-based algorithm; [2] *n* is the number of discordant samples per stage (according to VIDAS®-based classification). [3] According to the RT-PCR- and competitor ELISA-based algorithm (Table 3).

Table S5. Concordance of the VIDAS® dengue assays with the respective competitor ELISA, in the global population and per stage of DENV infection (common samples; see Figure S2)

VIDAS® dengue assay	Population	Positive Agreement		Negative Agreement		Overall Agreement	
		n/N [1]	% [95% CI]	*n/N* [1]	% [95% CI]	*n/N* [1]	% [95% CI]
**NS1**	Total	135/170	79.4% [72.7-84.8]	963/968	99.5% [98.8-99.8]	1098/1138	96.5% [95.2-97.5]
	Acute (all)	135/170	79.4% [72.7-84.8]	43/43	100.0% [91.8-100.0]	178/213	83.6% [78.0-87.9]
	Acute (D0-5)	89/98	90.8% [83.5-95.1]	20/20	100.0% [83.2-100.0]	109/118	92.4% [86.1-95.9]
	Post-Acute	N/A	-	123/123	100.0% [97.0-100.0]	N/A	-
	Recovery	N/A	-	621/626	99.2% [98.1-99.7]	N/A	-
**IgM**	Total	213/266	80.1% [74.9-84.4]	686/872	78.7% [75.8-81.3]	899/1138	79.0% [76.5-81.3]
	Acute (all)	120/142	84.5% [77.7-89.5]	57/71	80.3% [69.6-87.9]	177/213	83.1% [77.5-87.5]
	Acute (D0-5)	65/74	87.8% [78.5-93.5]	34/44	77.3% [63.0-87.2]	99/118	83.9% [76.2-89.4]
	Post-Acute	92/123	74.8% [66.5-81.6]	N/A	-	N/A	-
	Recovery	N/A	-	477/626	76.2% [72.7-79.4]	N/A	-
**IgG**	Total	811/948	85.5% [83.2-87.6]	164/190	86.3% [80.7-90.5]	975/1138	85.7% [83.5-87.6]
	Acute (all)	145/199	72.9% [66.3-78.6]	8/14	57.1% [32.6-78.6]	153/213	71.8% [65.4-77.4]
	Acute (D0-5)	91/110	82.7% [74.6-88.7]	2/8	25.0% [7.1-59.1]	93/118	78.8% [70.6-85.2]
	Post-Acute	99/123	80.5% [72.6-86.5]	N/A	-	N/A	-
	Recovery	567/626	90.6% [88.0-92.6]	N/A	-	N/A	-

[1] *n/N* is the ratio of the number of samples for which VIDAS® assays are in agreement (positive, negative and overall) with the competitor ELISA (reference test) to the number of samples tested either positive or negative (and overall) with the competitor ELISA. Abbreviations: CI, confidence interval; D0-5, 0-5 days post symptom-onset; N/A, not applicable.

Table S6. Concordance of the VIDAS® dengue assays with the respective competitor ELISA, according to time intervals post symptom onset.

		Positive Percent Agreement		Negative Percent Agreement		Overall Percent Agreement	
VIDAS® dengue assay	Time interval post symptom onset	*n/N* [1]	% [95% CI]	n/N [1]	% [95% CI]	*n/N* [1]	% [95% CI]
NS1	0 – 3 days	79 / 84	94.05% [86.81 ; 97.43] %	388 / 392	98.98% [97.41 ; 99.72] %	467 / 476	98.11% [96.44 ; 99.13] %
	4 – 5 days	49 / 54	90.74% [80.09 ; 95.98] %	172 / 174	98.85% [95.91 ; 99.86] %	221 / 228	96.93% [93.78 ; 98.76] %
	6 – 8 days	55 / 77	71.43% [60.51 ; 80.31] %	377 / 379	99.47% [98.11 ; 99.94] %	432 / 456	94.74% [92.29 ; 96.44] %
	9 – 15 days	4 / 7	57.14% [25.05 ; 84.18] %	71 / 71	100.00% [94.94 ; 100.00] %	75 / 78	96.15% [89.17 ; 99.20] %
	16 days – 1 month	0 / 3	0.00% [0.00 ; 70.76] %	31 / 31	100.00% [88.78 ; 100.00] %	31 / 34	91.18% [77.04 ; 96.95] %
	> 1 month	0 / 0	. [0.00 ; 0.00] %	16 / 16	100.00% [79.41 ; 100.00] %	16 / 16	100.00% [79.41 ; 100.00] %
	Unknown	4 / 4	100.00% [39.76 ; 100.00] %	3 / 3	100.00% [29.24 ; 100.00] %	7 / 7	100.00% [59.04 ; 100.00] %
IgM	0 – 3 days	39 / 46	84.78% [71.78 ; 92.43] %	300 / 380	78.95% [74.57 ; 82.75] %	339 / 426	79.58% [75.49 ; 83.13] %
	4 – 5 days	37 / 41	90.24% [77.45 ; 96.14] %	110 / 157	70.06% [62.49 ; 76.68] %	147 / 198	74.24% [67.73 ; 79.83] %
	6 – 8 days	126 / 164	76.83% [69.80 ; 82.63] %	193 / 233	82.83% [77.47 ; 87.13] %	319 / 397	80.35% [76.16 ; 83.96] %
	9 – 15 days	9 / 11	81.82% [52.30 ; 94.86] %	49 / 60	81.67% [70.08 ; 89.44] %	58 / 71	81.69% [71.15 ; 88.98] %
	16 days – 1 month	0 / 0	. [0.00 ; 0.00] %	25 / 31	80.65% [63.72 ; 90.81] %	25 / 31	80.65% [63.72 ; 90.81] %
	> 1 month	1 / 1	100.00% [2.50 ; 100.00] %	10 / 13	76.92% [49.74 ; 91.82] %	11 / 14	78.57% [52.41 ; 92.43] %
	Unknown	1 / 3	33.33% [6.15 ; 79.23] %	2 / 2	100.00% [15.81 ; 100.00] %	3 / 5	60.00% [23.07 ; 88.24] %
IgG	0 – 3 days	344 / 380	90.53% [87.16 ; 93.08] %	81 / 95	85.26% [76.77 ; 91.01] %	425 / 475	89.47% [86.39 ; 91.92] %
	4 – 5 days	174 / 200	87.00% [81.63 ; 90.97] %	22 / 27	81.48% [63.30 ; 91.82] %	196 / 227	86.34% [81.27 ; 90.21] %
	6 – 8 days	326 / 400	81.50% [77.40 ; 85.00] %	52 / 56	92.86% [83.02 ; 97.19] %	378 / 456	82.89% [79.17 ; 86.07] %
	9 – 15 days	52 / 66	78.79% [67.49 ; 86.92] %	9 / 12	75.00% [46.77 ; 91.11] %	61 / 78	78.21% [67.84 ; 85.92] %
	16 days – 1 month	27 / 30	90.00% [74.38 ; 96.54] %	4 / 4	100.00% [39.76 ; 100.00] %	31 / 34	91.18% [77.04 ; 96.95] %
	> 1 month	11 / 14	78.57% [52.41 ; 92.43] %	2 / 2	100.00% [15.81 ; 100.00] %	13 / 16	81.25% [56.99 ; 93.41] %
	Unknown	6 / 6	100.00% [54.07 ; 100.00] %	0 / 1	0.00% [0.00 ; 97.50] %	6 / 7	85.71% [48.69 ; 97.43] %

[1] *n/N* is the ratio of the number of samples for which VIDAS® assays are in agreement (positive, negative and overall) with the competitor ELISA (reference test) to the number of samples tested either positive or negative (and overall) with the competitor ELISA. Abbreviation: CI, confidence interval
